# New Appliances and Things Medical

**Published:** 1906-05-05

**Authors:** 


### NEW APPLIANCES AND THINGS MEDICAL.

### HÆMOSTASIN PARAGANGLIN.

(Burgoyne, Burbidges and Co., 12 and 16 Coleman Street, London, E.C.)

We have received from the above firm samples of two preparations containing the active principle of the supra-renal gland. Since the discovery of the important pharmacological properties of the active principle of this gland its field of therapeutic usefulness has been slowly but surely extending, and to-day it is known that many pathological states are benefited by the supra-renal glandular substance, in which its value must be regarded to some extent at least as empirical. Chief among these are those two conditions for which in the pamphlet before us paraganglin is specially recommended, namely, neurasthenia and visceral atony, including constipation. Paraganglin in the hands of Continental clinicians has certainly given in these affections most satisfactory results. Hæmostasin is the name given by Messrs. Burgoyne, Burbidges and Co. to their own preparation of the active constituent of the supra-renal gland. The solution before us contains this in the proportion of 1 in 1,000, and even diluted still 100 times possesses undoubtedly active hæmostatic properties. The greater necessarily includes the less, and hæmostasin is useful for all those affections in which paraganglin is of benefit. We think that the nomenclature of the preparations of the supra-renal gland is becoming unnecessarily complicated, but that there is no doubt whatever that both paraganglin and hæmostasin are active and reliable.

### CHIVERS' BOTTLED FRUIT.

(Messrs. Chivers and Sons, Limited, Histon, Cambridgeshire.)

We have received a sample of bottled fruit from this well-known firm. Formerly almost the entire supply of bottled fruits, which, on account of their dietetic value, are much in demand during the winter in this country, came from abroad. Thanks, however, to the energy and highly developed technique of Messrs. Chivers, an increasing quantity of this commodity is now coming from their fruit farm in Cambridgeshire. The samples of their produce which we have had the opportunity of tasting were in excellent condition, and retained largely the flavour of the fresh fruit; they were also entirely free from all objectionable properties often associated with goods of this class. The fruit used by this firm is derived from their own fruit farm, and as a consequence can be gathered under the most favourable conditions for bottling. We think these freshly preserved fruits are highly to be recommended, and form a most hygienic as well as pleasant substitute for fresh fruit during the winter months, or indeed whenever and wherever fresh fruit is difficult or impossible to obtain.

### ASEPTULES OF TRYPSIN.

(Oppenheimer, Son, and Co., 179 Queen Victoria Street, London, E.C.)

The recent discovery that trypsin is efficacious in the treatment of certain forms of malignant disease when injected *sub cutem*, or by the intra-muscular method, has naturally produced a demand for a sterile preparation of this substance. Messrs. Oppenheimer and Co. have succeeded in putting on the market an exceedingly active and sterile preparation of trypsin in a most convenient form. They supply it in tubes each containing ¼ grain of trypsin, or sufficient for one injection. The tubes are just broken and the liquid contents aspirated into a hypodermic syringe, and then injected in the usual way. These tubes have been given the convenient and appropriate name of aseptules. We have been able to convince ourselves that their contents are both active and sterile. We know of no more convenient form of trypsin for hypodermic or intra-muscular injection on the market, and are quite sure that this preparation will be appreciated by the profession as meeting a considerable demand.

